# “Serial” effects in parallel models of reading

**DOI:** 10.1016/j.cogpsych.2012.01.002

**Published:** 2012-06

**Authors:** Ya-Ning Chang, Steve Furber, Stephen Welbourne

**Affiliations:** a Neuroscience and Aphasia Research Unit (NARU), University of Manchester, Manchester M13 9PL, UK; b School of Computer Science, University of Manchester, Manchester M13 9PL, UK

**Keywords:** Reading, PDP, VWFA, Visual word recognition, Length effect, Computational modelling

## Abstract

There is now considerable evidence showing that the time to read a word out loud is influenced by an interaction between orthographic length and lexicality. Given that length effects are interpreted by advocates of dual-route models as evidence of serial processing this would seem to pose a serious challenge to models of single word reading which postulate a common parallel processing mechanism for reading both words and nonwords (Coltheart, Rastle, Perry, Langdon, & Ziegler, 2001; Rastle, Havelka, Wydell, Coltheart, & Besner, 2009). However, an alternative explanation of these data is that visual processes outside the scope of existing parallel models are responsible for generating the word-length related phenomena (Seidenberg & Plaut, 1998). Here we demonstrate that a parallel model of single word reading can account for the differential word-length effects found in the naming latencies of words and nonwords, provided that it includes a mapping from visual to orthographic representations, and that the nature of those orthographic representations are not preconstrained. The model can also simulate other supposedly “serial” effects. The overall findings were consistent with the view that visual processing contributes substantially to the word-length effects in normal reading and provided evidence to support the single-route theory which assumes words and nonwords are processed in parallel by a common mechanism.

## Highlights

► We develop a parallel reading model that includes a visual processing component. ► We examine whether the model can account for “serial” effects found in normal reading. ► The length by lexicality interaction is an emergent property of parallel models with visual processing. ► “Serial” effects in reading can be produced by parallel models. ► Visual processing may be the key to understanding “serial” effects in normal reading.

## 1 Introduction

Word length is one of the key diagnostic lexical variables to affect response latencies in visual word recognition. A considerable number of studies have examined word-length effects, either by using naming tasks or lexical decision tasks (Balota, Cortese, Sergent-Marshall, Spieler, & Yap, 2004; Forster & Chambers, 1973; Frederiksen & Kroll, 1976; New, Ferrand, Pallier, & Brysbaert, 2006; Weekes, 1997; Whaley, 1978). Forster and Chambers (1973) found significant word-length effects in both tasks where longer words tended to take a longer time to process than short words. However, a subsequent study conducted by Frederiksen and Kroll (1976) only obtained the equivalent word-length effects for both words and nonwords in naming; no effect was observed in the lexical decision task. Weekes (1997) found significant word-length effects for naming nonwords and weak effects for low-frequency words, but no length effects for high-frequency words. In his study, all items were monosyllables matched for number of phonemes, initial phoneme, orthographic neighbourhood size, number of enemies and friends, summed bigram frequency and average grapheme frequency to exclude potentially contaminating effects. In addition to these factorial experiments, a number of recent studies have been conducted to explore factors that influence lexical processing based on a large database of behavioural data-the English Lexicon Project (ELP) (Balota et al., 2004, 2007; New et al., 2006). These studies analyse the database by using multiple regression which avoids the difficulty in controlling for multiple-lexical variables that is often encountered in the factorial design. Balota et al. (2004) reported that the word-length was a significant predictor of response times and that the effect was much greater for the naming task than for lexical decision. The effect was present for both young and old groups tested on 2428 monosyllabic words. Their results identified length effects in word naming tasks and also a length by lexicality interaction in both naming and lexical decision tasks. One surprising aspect of these data demonstrated by New et al. (2006) was that a U-shaped pattern of word-length effects became apparent when they tested the performance of lexical decision across the full ELP dataset of over 33,006 words. A facilitated length effect was observed for words with 3–5 letters, with no significant length effect for words with 5–8 letters and an inhibited length effect for words with 8–13 letters. New et al. (2006) suggested that the inhibitory length effect for long words might be attributable to the decrease of visual acuity necessary to accommodate longer visual stimuli, also long words were likely to be refixated during reading.

In addition to the behavioural data, there is also evidence of word-length effects from neuroimaging studies. Hauk and Pulvermuller (2004) investigated the effects of word length and word frequency in a lexical decision task by using electroencephalography (EEG). Their results demonstrated that the long words and short words had the strongest event-related potentials (ERPs) in different time ranges. No interaction between word length and word frequency was observed in their study. However, Penolazzi, Hauk, and Pulvermuller (2007) demonstrated an interaction between word frequency and word length early in the ERP from 120 ms to 180 ms. Wydell, Vuorinen, Helenius, and Salmelin (2003) investigated the neural correlates of the interaction between word length and lexicality by using magnetoencephalography (MEG). They found reliable length effects for both words and nonwords in the occipital cortex at about 100 ms after the stimulus onset but words showed a smaller effect than nonwords in the left superior temporal cortex between 200 ms and 600 ms.

When all of this evidence is considered it seems inescapable that there are real effects of word length in naming both words and nonwords and that there is an interaction between word lexicality and word length even for monosyllabic words. The word-length effects for nonwords are greater than low-frequency words (Richards & Heller, 1976; Weekes, 1997), and the length effects for low-frequency words are greater than high-frequency words (Balota et al., 2004; Penolazzi et al., 2007; Weekes, 1997).

Many authors of reading models have considered that the word-length effect reveals important aspects of the natural reading process, which provide useful constraints on model design (Coltheart et al., 2001; New et al., 2006; Plaut, McClelland, Seidenberg, & Patterson, 1996; Weekes, 1997). Hence the presence or absence of the word-length effect for different types of stimuli should have important implications for models of single word reading and in particular on the debate between dual-route models (Coltheart, Curtis, Atkins, & Haller, 1993; Coltheart et al., 2001) (with a serial processing component) and connectionist models (Plaut et al., 1996; Seidenberg & McClelland, 1989), which are parallel processors. According to the dual-route theory normal readers have two different routes for converting print to speech (Coltheart et al., 1993). The lexical route operating in parallel accesses the lexical entry from the presented word form and retrieves its pronunciation for reading aloud. The non-lexical route, or the grapheme–phoneme conversion route, converts the graphemes of a letter string into phonemes by a set of grapheme–phoneme correspondence (GPC) rules in a sequential manner. The dual-route cascaded (DRC) model developed by Coltheart et al. (1993, 2001) is a computational realisation of the dual-route theory. In this model the lexical route is mainly used for processing regular and exception words, while the non-lexical route is needed to process nonwords, which it does in a serial manner processing the output phonology one phoneme at a time. This neatly explains the length × lexicality interaction as the nonwords (which show a strong length effect) are processed exclusively via a serial processing module, whereas the real words are supported largely by a parallel process. Thus, it is not difficult to imagine that the DRC model which postulates two different routes for processing words and nonwords can account for the differential word-length effects found by Weekes (1997). Indeed, Coltheart et al. (2001) tested the DRC model on Weekes’s dataset and demonstrated that the DRC model can reproduce the pattern of length effects found in the empirical data while Harm & Seidenberg, 1999, 2004; Plaut et al.’s (1996) parallel model failed to show the correct pattern of word length by word lexicality interaction. As a result of this the lexicality by word-length interaction in normal reading is often taken as a strong support for the dual-route theory. However, this conclusion is based on a presupposition that all connectionist models of reading aloud will fail to simulate length effects in normal reading.

An alternative view is articulated in connectionist models of reading (Harm & Seidenberg, 1999, 2004; Plaut et al., 1996; Seidenberg & McClelland, 1989) which assume that a single parallel processing mechanism is responsible for reading both words and nonwords. Seidenberg and McClelland (1989) proposed a general framework for the parallel distributed processing (PDP) model of word recognition and naming – hereafter known as SM89 model. The SM89 model has successfully reproduced a number of phenomena observed in normal and impaired reading. However, the main weakness of SM89 model is that the performance of SM89 model on reading nonwords was significantly poorer than the performance achieved by normal readers (Besner, 1989; Coltheart et al., 1993). In support of single-route theory, Plaut et al. (1996) developed a revised model, hereafter PMSP96, to improve the poor performance of SM89 model on nonword reading. The PMSP96 model showed a remarkable improvement in nonword reading such that it reads nonwords as well as normal readers. The key difference between the PMSP96 and the SM89 models relates to the way in which orthographic and phonological representations were encoded. The PMSP96 encoding scheme was designed to facilitate maximum use of the regularities in the mappings between orthographic and phonological representations. To do this each letter string was parsed into the onset, vowel and coda clusters of graphemes and phonemes. By this scheme, it is possible to construct position independent orthographic and phonological representations according to graphotactic and phonotatctic constrains, allowing spelling–sound regularities among words to be captured effectively. More recently, Monaghan and Ellis (2010) showed that orthographic length was a significant predictor for the naming latencies produced by their parallel model which mapped orthography to phonology. However, as their study was not designed to investigate the word-length effect, they did not perform a regression analysis on nonwords. It remains unclear whether their model can produce the length effect for nonwords and show a word length by lexicality interaction. Despite the fact that the parallel models have proven to account for empirical data obtained from both normal and impaired reading, they cannot currently capture the patterns of the length effects that are seen in human data (e.g., Weekes, 1997). In addition, there are two other length related phenomena, which are often used as arguments against parallel models: the position of irregularity effect (Rastle & Coltheart, 1999) and the whammy effect (Rastle & Coltheart, 1998). The position of irregularity effect in reading is found in low frequency exception words where the regularity effect is modulated by position. This effect has been interpreted as evidence for serial processing. However, Zorzi’s (2000) CDP model was able to capture this effect due to the fact that the position of irregularity factor is actually confounded with grapheme–phoneme consistency. The whammy effect relates to the fact that 5-letter nonwords with 3 graphemes produce longer latencies than 5-letter nonwords with 5 graphemes. According to serial processing accounts, the spurious phoneme unit will activate early inhibiting the late activation of the correct phoneme. However, the whammy effect may also be explained by the parallel processing account simply due to competition between possible target phonemes, which is greater in 3 grapheme words. The existence of these “serial” length related effects means that parallel models could be vulnerable to the criticism that without some kind of additional serial processing component they may never be able to account adequately for these phenomena (Coltheart et al., 2001; Rastle et al., 2009; Weekes, 1997).

In addition to the triangle and DRC models some hybrid reading models have begun to emerge, which attempt to combine the strengths and eliminate the weaknesses of the two approaches. The connetionist dual process (CDP) model developed by Zorzi, Houghton, and Butterworth (1998) has the dual-route architecture but is based on parallel processors. In the CDP model, the lexical route is conceptualised as having a mediated hidden layer (i.e., the lexicon layer) between orthographic and phonological representations. As for the non-lexical route, it has direct connections between orthographic and phonological layers to capture the regularities of spelling–sound relationships in English and it operates in parallel. The CDP model was also tested for length effects by Coltheart et al. (2001) and the results showed no interaction between length and lexicality. It seems that CDP model can at best only produce a weak length effect for nonwords (Perry, Ziegler, & Zorzi, 2007; Zorzi, 2010). A more sophisticated model named CDP+, was subsequently developed by Perry et al. (2007) to address this weakness. The CDP+ model combines the non-lexical route of CDP model with the lexical route of DRC model. It is important to note that the non-lexical route of CDP+ has an addition of a serial grapheme parsing mechanism, which is a major difference from CDP. This procedure generates a length dependent effect because it parses letter strings into graphemes sequentially from left to right. Once parsed the available graphemes are instantaneously used as input for the mappings between orthography and phonology. The phonological processing time is influenced by this serialising procedure. The CDP+ model is therefore a parallel model which has a serial processing component. Perry et al. (2007) tested their model on Weekes (1997) data and the results showed the CDP+ model produced a length effect and a correct interaction between length and lexicality as seen in human data. According to Perry et al. (2007) and Zorzi (2010), the addition of the serialising procedure was essential to capture the length effect.

On this basis the length effect still appears to be a critical issue for any parallel reading model which does not utilise a serial processing (Rastle et al., 2009). However, the assumption that length effects are the hallmark of serial processing components remains arguable. Proponents of the triangle model have made an attempt to provide alternative explanations of this issue. Seidenberg and Plaut (1998) argue that word length is complicated variable to understand because it affects several processes of word reading including the encoding of the visual display and the production of articulatory output. More recently, Kawamoto (2010) also propose similar ideas that other factors such as limited visual processing capacity and articulatory processing could be responsible for the length effect. These aspects are outside the domain of existent triangle models and so have not yet been tested. Articulatory processing may contribute to the length effect because there are more complicated initial phoneme clusters for long words than short words (e.g., /spr/ for SPRAY, /r/ for RAY). This might result in disproportionate delay times for long words to be articulated (Kawamoto & Kello, 1999; Plaut, 1999). Based on this evidence it would seem at least a proportion of length effects can be accounted for by the articulatory process. However, this is unlikely to be the whole story because the differential length effects demonstrated by Weekes (1997) must be largely independent of the articulatory characteristics as the stimuli are carefully matched for initial phoneme.

There may also be some basic perceptual factors that make a substantial contribution to length effects in normal reading. This view is supported by consideration of the natural constraints of visual processing in visual word recognition. Visual acuity has been shown to decrease as letters are moved further from the centre of fixation, resulting in the loss of visual information. The drop of visual acuity is approximately linear with the distance from the fixation location (Nazir, Oregan, & Jacobs, 1991). Hence, long words tend to receive more re-fixations than short words to overcome the limits of visual acuity, suggesting another possible account of the word-length effect. On the other hand, word length seems to have relatively little influence in skilled readers in compared with beginning readers or children as the skilled readers are able to maximise visual acuity by the selection of fixation location, known as the ‘optimal viewing position’ (OVP) (McConkie, Kerr, Reddix, Zola, & Jacobs, 1989; Oregan, Levyschoen, Pynte, & Brugaillere, 1984). For English readers, performance is the best when the words are fixed between the beginning and the middle of the words across different visual recognition tasks. The most likely explanation for the OVP effect is perceptual learning (Brysbaert & Nazir, 2005; Nazir, 2000; Nazir, Ben-Boutayab, Decoppet, Deutsch, & Frost, 2004). Over learning, the representations of word stimuli may be rebuilt repeatedly for different retinal locations to form the visual pattern memories. It is likely that the best viewing location for the rapid and efficient recognition of a word can be optimised through the exposure to multiple representations of a particular word. Interestingly, Nazir et al. (2004) found the performance of word recognition was highly correlated with the distribution of landing positions of the eyes in words; this result was observed only with familiar word stimuli but not with unfamiliar nonwords, suggesting the role of low-level perceptual learning in visual word recognition of these familiar stimuli.

Another stream of behavioural evidence comes from studies where word-length effects are artificially induced by adding visual distortions to stimuli during reading. The nature of the distortion can be very varied such as reducing stimulus contrast (Fiset, Arguin, & Fiset, 2006; Legge, Ahn, Klitz, & Luebker, 1997), displaying stimulus in normal peripheral vision (Legge, Mansfield, & Chung, 2001), displaying stimulus in the left visual field (Bub & Lewine, 1988; Ellis, Young, & Anderson, 1988; Lavidor & Bailey, 2005; Lavidor & Ellis, 2002; Young & Ellis, 1985), displaying stimulus vertically (Bub & Lewine, 1988; Cohen, Dehaene, Vinckier, Jobert, & Montavont, 2008) or increasing letter spacing of the word stimulus (Cohen et al., 2008). All of these stimulus manipulations produce a certain amount of visual difficulties in normal readers that leads to the emergence of the word-length effect. These findings from psychophysical research suggest that the word-length effects can be triggered by any form of unfamiliar visual stimuli. From this perspective we would argue that the characteristic length effects found when reading nonwords might result from the unfamiliarity of the visual stimuli rather than being evidence of any special serial processing mechanism. Although we acknowledge that in some circumstances the unfamiliarity maybe so great as to prompt a completely different sequential strategy that is unrelated to normal reading mechanisms (e.g. for reading mirror image text).

In addition to evidence from normal readers it is important to consider evidence from patients who suffer from a disorder known as pure alexia (PA). Patients with pure alexia are characterised by slow reading behaviour and reading times that are linearly correlated with word length (Patterson & Kay, 1982; Warrington & Shallice, 1980). Many researchers have argued that the locus of this deficit is at the early stages of visual processing (Arguin, Fiset, & Bub, 2002; Behrmann, Nelson, & Sekuler, 1998; Behrmann & Shallice, 1995; Farah & Wallace, 1991; Kay & Hanley, 1991; Rapp & Caramazza, 1991). Under this view, which we share, the exaggerated word-length effects observed in PA patients are thought to result from a visual deficit, although the underlying cause of pure alexia remains somewhat debatable (Coslett & Saffran, 1989; Warrington, 1980). The idea that there is a visual perception deficit in pure alexia was first proposed by Farah and Wallace (1991). This view is supported by evidence that PA patients have the impaired performance on both linguistic and non-linguistic stimuli (Behrmann et al., 1998; Mycroft, Behrmann, & Kay, 2009). Many recent functional imaging studies suggest that pure alexia results from damage to a specific neural region of left hemisphere, known as the visual word form area (VWFA) (Cohen et al., 2004). The VWFA is often regarded as an area which generates an invariant representation of letter identities, which is in the mid-fusiform gyrus within the left occipito-temporal sulcus. The activations of the VWFA are modulated by word lexicality, with stronger activations for nonwords than for words (Fiez, Balota, Raichle, & Petersen, 1999; Price, Wise, & Frackowiak, 1996; Xu et al., 2001). More recently, Schurz et al. (2010) showed an interaction between word length and lexicality in the VWFA. The length effect for words was only observed in occipital cortex but not in the VWFA, while the length effect for nonwords was obtained throughout the ventral visual pathway including the VWFA. Despite the considerable evidence for the involvement of the VWFA in visual word recognition, it seems unlikely to be specialised for visual word forms (Devlin, Jamison, Gonnerman, & Matthews, 2006; Price & Devlin, 2003). Rather, Price and Devlin (2003) argued that left mid-fusiform gyrus (VWFA) was a polymodal area driven mostly by visual input. They supported this by showing that VWFA was not only activated by word form stimuli but also activated when subjects were asked to name objects and repeat auditorially presented words.

In summary, there is good evidence to support the view that the differential length effects for monosyllabic words and nonwords in normal reading may arise as a consequence of visual processing procedures occurring in the occipital mid-fusiform gyrus. The characteristic lexicality by length interaction could stem from the greater visual difficulty with unfamiliar stimuli resulting in a longer visual processing time for nonword reading and a more marked dependence on word length. From this viewpoint, it seems reasonable that existing parallel models cannot provide an adequate account of this interaction without the implementation of some kind of visual system. To test this, the current study developed a parallel model, including a visual processing stage, to investigate whether this additional visual factor can account for the word-length effects in normal reading. The key test of this was the ability of the model to replicate the lexicality by length interaction found by Weekes (1997). Four simulations were conducted: Simulation 1 used fixed orthographic representations adopted from PMSP96 model. Simulation 2 used learned orthographic representations (i.e., the orthographic representations were allowed to emerge during training). Simulation 3 included an added semantic influence on phonological outputs. Simulation 4 extended the work by exploring the effect of different viewing positions and by testing whether the model could simulate other “serial effects”.

## 2 Simulation 1

The model was developed on the basis of the general framework of the triangle model (Plaut et al., 1996; Seidenberg & McClelland, 1989), but with the addition of a visual input layer and an additional hidden layer prior to the orthographic layer, which formed the input in the original versions of the triangle models. The training corpus consisted of 8160 monosyllabic words including Plaut et al.’s (1996) word list, Weekes’ (1997) word list, and all the monosyllabic words (including inflections) from the ELP database except for prefixes, suffixes and proper nouns. The performance of the model was first compared with the performance achieved by Plaut et al.’s (1996) model, and then the model was tested on Weekes’ (1997) dataset. The ability of the model to account for the differential length effects on naming words and nonwords was assessed.

### 2.1 Method

#### 2.1.1 Model architecture

**Fig. 1:**
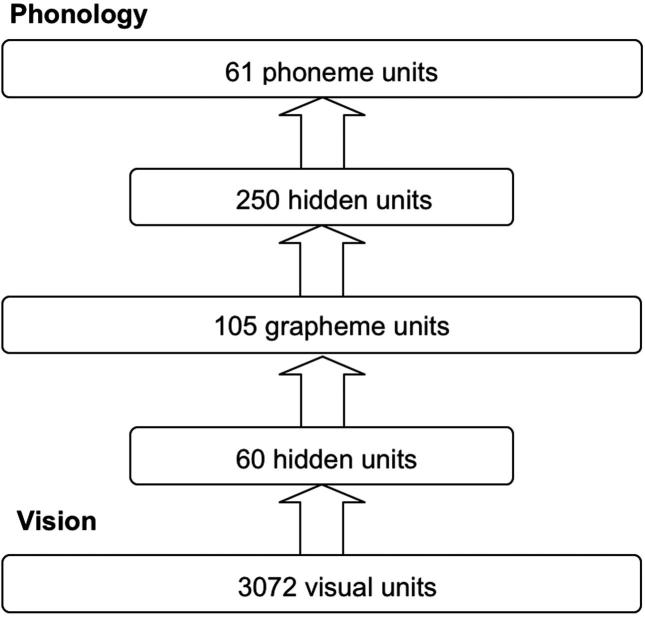
The architecture of the feedforward network. The arrows represent fully connections from one layer to another layer.

Fig. 1 shows the architecture of the network. The network consisted of five layers of units: 3072 visual units, 60 hidden units, 105 grapheme units, 250 hidden units and 61 phoneme units. The first two layers of the network formed the visual processing system, and the last three layers of the network were for the mapping from the orthographic to phonological representations in which the function was exactly the same as the feedforward network in the Plaut et al.’s study (1996, Simulation 1). Each layer of units was fully connected to the next layer. Each visual unit was connected to each unit at the hidden layer above, and each hidden unit was connected to each unit at the orthographic layer. Similarly, each orthographic unit was connected to each unit in the hidden layer above and each hidden unit was connected to each unit in the phonology layer.

#### 2.1.2 Visual image representations

**Fig. 2:**
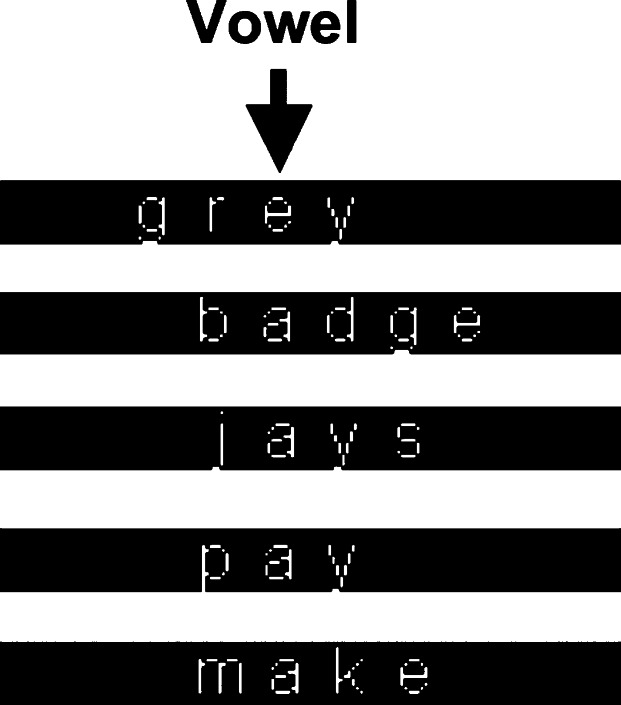
Examples of lower case, 12-points words in Arial font in the training corpus. The vowel of each word is aligned on the sixth column.

The network was directly fed with bitmap images of words. The 12-point lower case words in Arial font were represented in white against a black background. Each word was positioned with its vowel aligned on a fixed column of the image. (Networks using other types of visual representations are explored in Section 6.1.) Twelve columns were used in all and the size of each column was 16 × 16 pixels. Hence, there were in total 3072 visual units for the representations of input images. Fig. 2 shows examples of word images used for the training. All the word images were created by using the Matlab programming software, and image processing tools were used to convert word images into input binary values for visual representations.

#### 2.1.3 Orthographic and phonological representations

The orthographic and phonological representations schemes were taken from the PMSP96 model (Plaut et al., 1996). For the orthographic representations, each word as a letter string was parsed into the onset, vowel and coda clusters of graphemes. Similarly, for the phonological representations the word was also parsed into the onset, vowel and coda clusters of phonemes. Table 1 shows the orthographic and phonological representation schemes used in the simulation.

#### 2.1.4 Training procedures

The network was trained using the back-propagation algorithm with a global learning rate of 0.05, a weight decay of 0.00001 and momentum of 0.95. Cross entropy was used as the error function. The initial weights for the connections between units were set to random values between −0.1 and 0.1. The training corpus consisted of 8160 word image bitmaps. The network was then trained to generate the orthographic and phonological outputs based on these visual representations. Frequency was implemented by using a square-root function to compress the frequency range (Plaut et al., 1996; Seidenberg & McClelland, 1989). This compressed frequency was used to scale error derivatives for the computation of back-propagation. To preclude the possibility that the simulation results could be a consequence for one particular set of initial weights, the network was trained 10 times with different random initial weights.

#### 2.1.5 Testing procedures

The decoding procedure for reading the orthographic output of the network was straightforward. For all 3 grapheme groups, the orthographic output was the ordered composition of all active graphemes in each group whose activations were greater than .5. Similarly, the procedure for the generation of the phonological output was determined according to the activities of the phoneme units at the phonology layer. However, as in Plaut et al. (1996) the procedure here was slightly more complicated. For the vowel units the most activated vowel unit was selected as output. For the onset and coda the output units were divided into groups of mutually exclusive units the highest active unit above .5 was taken as the output for each group. If no unit was active above .5 than none of the group was included as part of the output. If either of the ks, ts or ps units was active along with their components than the order of the components was reversed. The performance of the network was tested every 200 epochs throughout the training. The key measure of the performance of the network comes from testing whether the network can produce correct pronunciation at the phonology units. It is possible that the network could make orthographic errors, but still produce the correct pronunciation. However, accuracies for both orthographic and phonological layers were recorded.

### 2.2 Results

After 1600 training epochs, the accuracy rates were 99.26% and 99.06% for the orthographic and phonological levels respectively. Table 2 shows the performance of the network compared with the performance achieved by human subjects and the PMSP96 model. As in PMSP96 the network made most errors on the 13 sets of homographs in training corpus. Homographs have the same spelling but different pronunciations, and the appropriate one is usually dependent on the context used. Since the network has no access to context or semantic information, it is impossible for it to distinguish words with the same spelling but different meanings – so performance on these items will be at chance levels. To make a useful comparison with the PMSP96 model we also tested on a restricted word list corresponding to the training corpus of PMSP96. On these words the network achieved accuracy rates of 99.57% and 99.07% at the orthographic level and the phonological level respectively. This was very close to the 99.13% phonological accuracy achieved by the PMSP96 model. For nonword reading, the network was tested on a list of 43 regular consistent nonwords taken from Glushko (1979). The consistent nonwords were created by changing the onset of an existing regular word. As shown in Table 2, the performance of the network at the phonological output was almost indistinguishable from that of the PMSP96 model, but 4.34% more accurate than the human subjects’ performance.

#### 2.2.1 Frequency and consistency effects

**Fig. 3:**
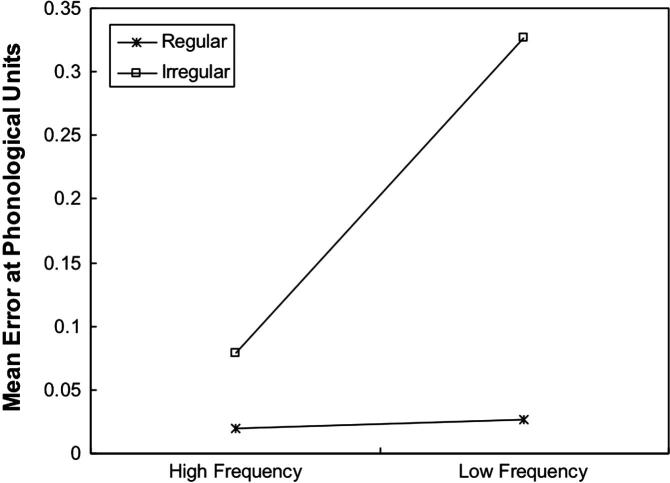
Mean phonological error as a function of frequency and consistency across 10 runs for Simulation 1.

In addition to testing the network on normal reading and nonword reading, it is important to verify whether the network could replicate the basic effects of frequency and consistency in naming latency (Baron & Strawson, 1976; Glushko, 1979; Jared, McRae, & Seidenberg, 1990; Plaut et al., 1996; Seidenberg & McClelland, 1989; Stanovich & Bauer, 1978). Just as in PMSP96 Simulation 1, error score was used as an analogue of naming latency. In the current simulation, the network was tested on four sets of test stimuli: high-frequency regular words, low-frequency regular words, high-frequency irregular words and low-frequency irregular words. Each set of stimuli consisted of 24 words taken from Taraban and McClelland (1987). All words were matched for written frequency. Fig. 3 shows the mean phonological error made by the network on words with different consistencies as the function of frequency. As can be seen in Fig. 3, the network had larger error scores when naming irregular words than regular words. There was also much larger frequency effect on naming irregular words than regular words. A 2 × 2 analysis of variance (ANOVA) was performed to analyse the data, where frequency and consistency were treated as between-group variables. The main effect of frequency was significant, *F*(1, 92) = 22.58, *p* < .001. The main effect of consistency was also significant, *F*(1, 92) = 44.74, *p* < .001. There was a significant interaction between frequency and consistency, *F*(1, 92) = 20.19, *p* < .001.

### 2.3 Simulation of word-length effects

The key objective for this study is to investigate whether in a computational model of reading that uses a single mechanism to process words and nonwords it is possible that a differential effect of word length may emerge within the visual processing stage of the model. The crucial test of this is the presence of a lexicality by length interaction such that word processing is minimally influenced by length whereas nonword processing is strongly length dependent (Weekes, 1997). There were 100 high-frequency words, 100 low-frequency words and 100 nonwords in the Weekes’s experiment. Within each lexical group, items were subdivided into 25 quartets of items, and each quartet of items consisted of 3, 4, 5 and 6 letter words. Items within each quartet were matched for their initial phonemes and initial letters. Each frequency grouping was also matched for mean orthographic neighbourhoodsize, mean number of phonemes, mean number of enemies, mean number of friends, summed bigram frequency, and mean grapheme frequency.

The network was tested on each lexical group. The results showed that the network could correctly read all high-frequency and low-frequency words. The naming performance of the network for the nonword stimuli was 99.3% and 96.8% at the orthographic level and the phonological level respectively. Fig. 4a shows the mean error score at the phonological units as a function of word length and word lexicality across 10 runs and Fig. 4b shows the pattern of the mean error score at the orthographic layer. Two 3 × 4 within-subjects ANOVA analyses were performed to analyse both the phonological and orthographic errors. For the repeated measure analysis of the phonological error, both the main effect of word type *F*(1.23, 11.11) = 464.68, *p* < .001 (with a Greenhouse–Geisser adjustment to compensate for the violation of the sphericity assumption) and the main effect of word length, *F*(1.94, 17.43) = 11.85, *p* = .001 (with a Greenhouse–Geisser adjustment to compensate for the violation of the sphericity assumption) were significant. The interaction between word type and word length was also significant, *F*(2.05, 18.41) = 11.38, *p* = .001 (with a Greenhouse–Geisser adjustment to compensate for the violation of the sphericity assumption). However, there was no trend of a linear effect of length either for words or for nonwords. Analysis of the mean error scores at the orthographic units revealed main effects for both word length, *F*(1.20, 10.79) = 139.79, *p* < .001 (with a Greenhouse–Geisser adjustment to compensate for the violation of the sphericity assumption) and word type, *F*(2, 18) = 67.49, *p* < .001. There was a significant word length and word type interaction *F*(1.82, 16.39) = 29.85, *p* < .001 (with a Greenhouse–Geisser adjustment to compensate for the violation of the sphericity assumption). As shown in Fig. 4b, there seems to be a trend towards a linear effect of length, but the interaction between words and nonwords is somewhat less marked than in Weekes’ data.

## 3 Simulation 2

In Simulation 1, the network was trained to map from vision to orthography, and then from orthography to phonology. This shows that merely adding a visual layer to the PMSP96 model is not sufficient to account for the length effects found in human reading. This may not be surprising, as this method of introducing visual processing to the triangle model is probably not realistic. In particular, the use of predefined orthographic representations cannot reflect the way in which children really learn to read. Phonological and semantic representations clearly exist in some form prior to reading acquisition, but orthographic representations must develop as part of the process of reading. This was not reflected in Simulation 1. Simulation 2 explored the effect of allowing the orthographic representations to be learned by using the same architecture as Simulation 1, but without applying orthographic targets to the orthographic layer of units. The performance of the network in Simulation 2 was examined in comparison with the results from Simulation 1.

### 3.1 Method

#### 3.1.1 Model architecture

**Fig. 5:**
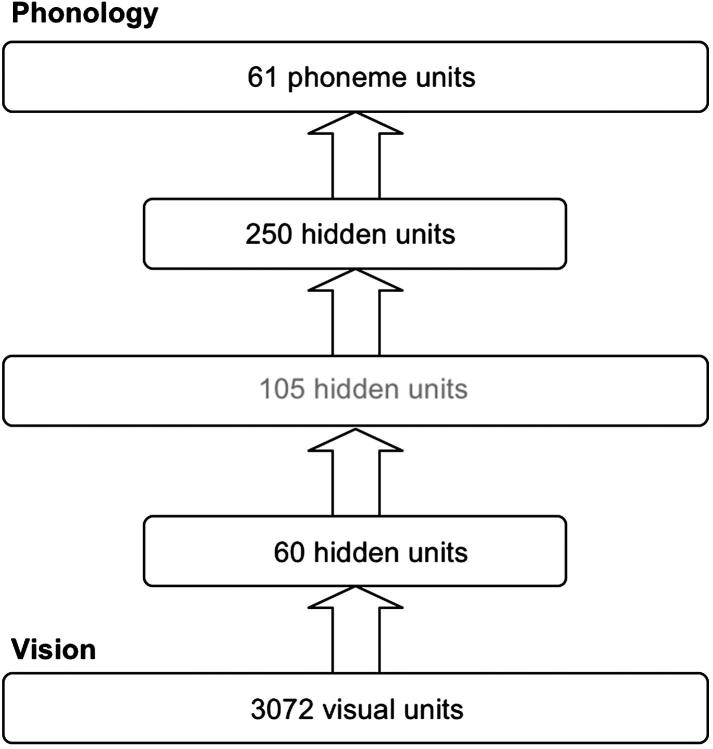
The architecture of the feedforward network with an additional hidden layer instead of an orthographic layer.

Fig. 5 shows the architecture for Simulation 2. The encoding procedures for visual and phonological representations were the same as those used in Simulation 1. The major change in the training procedure from Simulation 1 was that the network was trained to map from visual input to phonological output without being given the explicit knowledge of orthography. Other training conditions were exactly the same as those used in Simulation 1. The procedures for testing the performance of the network on naming words and nonwords were the same as in Simulation 1.

### 3.2 Results

After 1600 epochs of training, the network was 99.19% accurate. Most words not being pronounced correctly were very low-frequency words and homographs. For nonword reading, the network was again tested on the nonwords taken from Glushko (1979). The performance of the network was 93.25% which is closer to human performance than either PMSP96 or Simulation 1. Overall, the results showed the network even without implicit knowledge of orthography can be trained successfully to name words and nonwords.

#### 3.2.1 Frequency and consistency effects

**Fig. 6:**
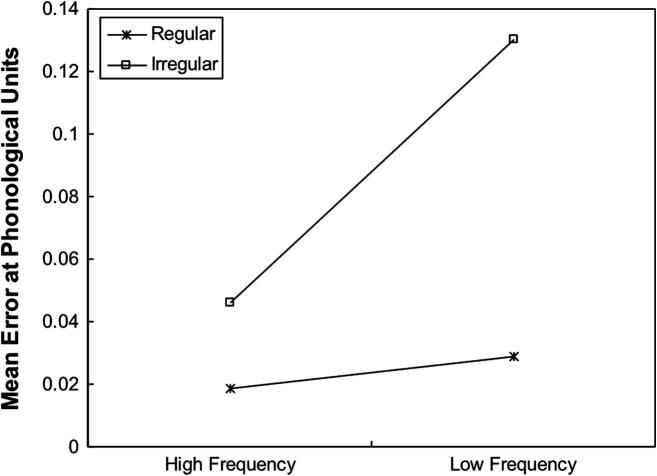
Mean phonological error as a function of frequency and consistency across 10 runs for Simulation 2.

Simulation 1 showed that the model can reproduce the interaction between frequency and consistency. It is also important to examine whether Simulation 2 can simulate this effect. Fig. 6 illustrates the significant interaction between frequency and consistency, *F*(1, 92) = 15.00, *p* < .001. The ANOVA analysis also showed that both main effects of frequency, *F*(1, 92) = 24.64, *p* < .001 and consistency, *F*(1, 92) = 46.26, *p* < .001 were significant.

### 3.3 Simulation of word-length effects

**Fig. 7:**
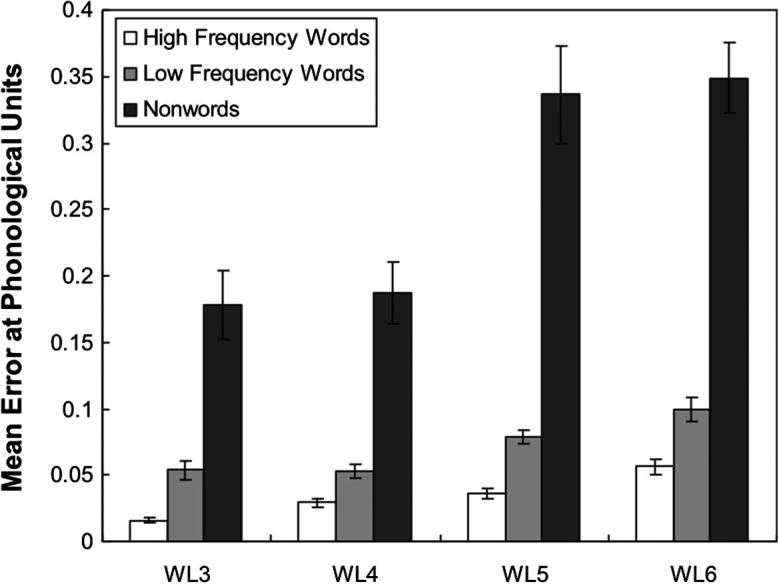
Mean error score computed at the phonological level for Simulation 2 as a function of word length and word type. Error bars represent standard errors.

The network was also tested on Weekes’ (1997) dataset. The results showed that the network can correctly read all high-frequency and low-frequency words. The performance on nonwords was at 92.60% correct. A 3 × 4 within-subjects ANOVA analysis was performed to analyse the error scores produced by the network. The main effects for both word type, *F*(1.04, 9.38) = 156.64, *p* < .001 (with a Greenhouse–Geisser adjustment to compensate for the violation of sphericity assumption) and word length, *F*(3, 27) = 19.86, *p* < .001 were significant. There was also a significant word length by word type interaction, *F*(2.07, 18.62) = 7.87, *p* = .003 (with a Greenhouse–Geisser adjustment to compensate for the violation of sphericity assumption). Fig. 7 shows the interaction between word length and word type. The pattern of this interaction was similar to that seen in Weekes’s study. For nonwords, the errors became larger as the number of letters increased while for words this increase was present but much reduced. One minor discrepancy between these findings and those of Weekes was that the word-length effect for high-frequency words was statistically reliable (though small) for the current simulation but not for the human subjects. To examine whether there is an interaction between word length and word frequency, a 2 × 4 within-subjects ANOVA analysis was performed. The result showed that both main effects for word length, *F*(3, 27) = 74.78, *p* < .001, and for word frequency, *F*(1, 9) = 134.28, *p* < .001, were significant. There was a significant interaction between word length and word frequency, *F*(3, 27) = 7.94, *p* = .001. A linear regression analysis was also performed to compare the regression coefficients for the different stimuli types, the coefficient for nonwords (slope = .069; CI: .000–.138) was much greater than that for both low-frequency words (slope = .016; CI: .004–.028) and high-frequency words (slope = .013; CI: .007–.018), indicating the word-length effect on nonword reading was greater than that for low-frequency words, and the word-length effect on naming low-frequency words was greater than that for high-frequency words.

## 4 What causes the word-length effects?

Both Simulation 1 and Simulation 2 could name words and nonwords correctly. However, only Simulation 2 produced a correct interaction pattern of word length and word type. The key difference between two networks relates to the orthographic representations. In Simulation 1 the network was explicitly trained to map from V → O → P, while in Simulation 2, the orthography layer was replaced with a hidden (H) layer of the same size (allowing the network to develop it’s own “orthographic” representations). In Simulation 1, as in PMSP96, the orthographic and phonological representations are designed specifically to overcome the dispersion problem: while this is generally a desirable thing as it promotes generalisation it is also likely to reduce any length effect as the orthographic representations become insensitive to length. For instance, graphemes “P” in PAY and “P” in SPRAY are coded exactly the same in the onset cluster irrespective of its letter position. However, in Simulation 2, there is no explicit orthographic layer in the network – this seems to result in the development of representations that have a degree of sensitivity to length. If this is true we would expect that the performance of a particular letter within a word might be dependent on the position of the letter in a word (e.g., letter “P” in PAY versus letter “P” in SPRAY), and this effect would be modulated by the frequency with which that letter appears in that position relative to the vowel. Table 3 shows the probability of location of individual letters within a word based on the statistics of the training corpus. The V-Pos here represents the fixed position on which the first vowel of each word aligned. The value for each letter in a particular position is the probability of the letter appearing in that position. (Note that this is weighted by the frequency of the whole word within the corpus.) As can be seen from Table 3, the probability of letter “P” in PAY appearing to L-Pos1 is 0.3535 while the probability of letter “P” in SPRAY appearing to L-Pos2 is 0.1904. We hypothesised that Simulation 2 could keep this kind of letter position information and should be more sensitive to the location of an individual letter within a word. Thus the performance on any individual letter/sound correspondence should be predicted by the probabilities of the letter location shown in Table 3. Given that the probability of encountering a particular letter decreases with increasing distance this is likely to produce a length effect. Specifically, the error made by Simulation 2 would increase as there were more letters in the periphery. By contrast, Simulation 1 should be less affected by the location of individual letter within a word.

### 4.1 Method

**Fig. 8:**
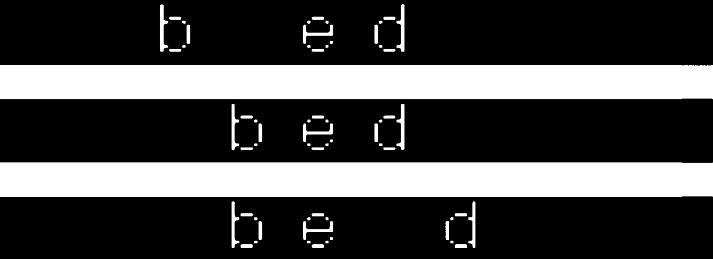
Examples of 3-letter CVC words: top – the initial consonant “b” is moved one slot away from vowel toward the left; middle – the trained condition; bottom – the final consonant “d” is moved one slot away from vowel toward the right.

To test this idea we used a set of all 3-letter high frequency CVC words in our training corpus. There were two tests: (1) whether the network could pronounce the initial consonants where they were moved one slot further from vowel toward the left; (2) whether the network could pronounce the final consonants moved one position to the right. Examples of the stimuli are shown in Fig. 8. Where this manipulation resulted in the initial or final consonant falling into a slot where it had never been presented before the stimuli was not included in the test. Hence, there were in total 68 words for the initial consonant test and 74 words for the final consonant test.

### 4.2 Results

Figs. 9a and 9b show the error score on phonology for the letter of interest within the word on the initial consonant test and final consonant test. The error score was for the individual letter rather than for whole word. As can be seen in Figs. 9a and 9b, the error increments in Simulation 2 were much larger than in Simulation 1 when the letter was moved. Two 2 × 2 repeated measures ANOVA with letter position (moved, not moved) as a within-subject factor and Simulation (1, 2) as a between-subject factor were performed to analyse the data. For the initial consonant test, the main effect of letter position was significant, *F*(1, 18) = 64.79, *p* < .001. There was a significant interaction between Simulation and letter position, *F*(1, 18) = 59.91, *p* < .001. Similarly, for the final consonant test, the main effect of letter position, *F*(1, 18) = 96.96, *p* < .001, was significant. There was a significant interaction between Simulation and letter position, *F*(1, 18) = 82.03, *p* < .001. These results demonstrated that Simulation 2 was more sensitive to the letter position within a word than Simulation 1 at the phonological level. The performance of Simulation 2 is consistent with the probability pattern shown in Table 3 in which letters being further from the middle of a word tend to have smaller probabilities to be seen before than letters being near the middle of a word.

## 5 Simulation 3

In Simulation 2, the network demonstrated a differential word-length effect similar to that seen in human subjects. The simulation data does not completely tally with Weekes’ (1997) data because there is a small word-length effect for high-frequency words, which Weekes did not find in human subjects. However, more recent work suggests there should be a small but reliable length effect for all words (Balota et al., 2004). Another possibility is that the reliable length effect on naming high-frequency words in Simulation 2 may be a consequence of the absence of semantic effect in the current model. In normal reading, the time course of phonological processing would be influenced by the meanings of words and it is likely that this influence will mitigate or even completely eliminate length effects for words. Simulation 3 examined to what degree the length effects for words will be reduced by semantic contributions.

In the literature, several schemes for representing semantic information have been proposed both for behavioural studies and for use in computational modelling (Garrard, Lambon Ralph, Hodges, & Patterson, 2001; Harm, 2002; Landauer, Foltz, & Laham, 1998; Lund & Burgess, 1996; McRae, Cree, Seidenberg, & McNorgan, 2005; Plaut, 1997; Rohde, Gonnerman, & Plaut, 2006), but no consensus has been reached on the best method to use. A full implementation of semantic representations is beyond the scope of the present paper; however, it is possible to circumvent the problem of semantic representation and still approximate the semantic effect by adding external input to the phoneme units at the phonological layer (Plaut et al., 1996; Welbourne & Lambon Ralph, 2007; Woollams, Lambon Ralph, Plaut, & Patterson, 2007). The assumption being that the semantic pathway has learned the meaning and pronunciation of a word and it would therefore have positive contributions to the activations of phonemes within the phonological pathway. In Simulation 3, the network was trained with the addition of this external semantic influence. Once trained it was tested on Weekes’ (1997) dataset to examine whether this manipulation altered the pattern of length effects for words and nonwords.

### 5.1 Method

The architecture of the network was the same as in Simulation 2 except that there was an additional semantic input directly to the phonological layer. To simulate the development of the semantic pathway in human reading system, the contribution of the semantic input to the phonological units was gradually increased with time and modulated by the frequency of the word. The value of the semantic input was set according to the following equation:

$$\text{Semantic input}=\frac{1}{1+e^{-(k(\log(f+2))-1)}}\times U(t)$$

where *f* was the frequency of the word (Kucera & Francis, 1967), and *k* was selected so that the most frequent word had a maximum semantic input of .95 and the least frequent word had a minimum semantic input of .5 (*k* = 1.14). *U*(*T*) was a time-dependent stepwise function, where each step was 200 training epochs. The magnitude of the stepwise function ranged from 0.2 to 1.6 in steps of 0.2; these parameters were adapted from those used in Welbourne and Lambon Ralph’s (2007) simulation.

### 5.2 Results

**Fig. 10:**
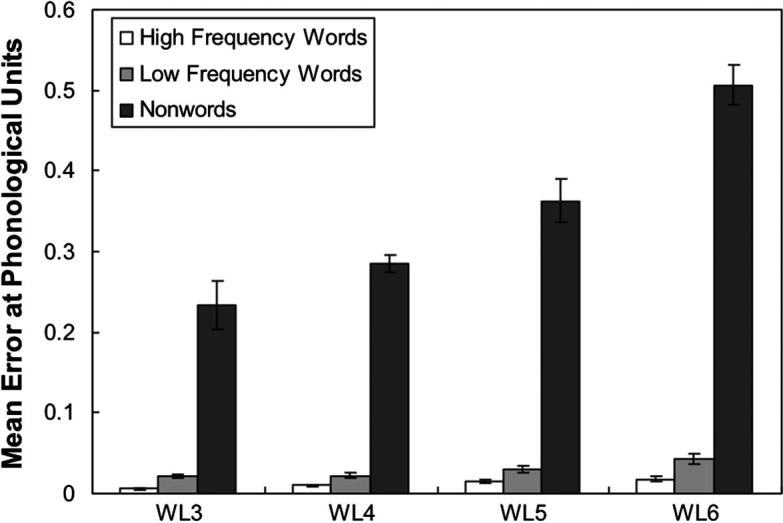
Mean error score computed at the phonological level for Simulation 3 as a function of word length and word type. Error bars represent standard errors.

After training, for word reading, the network achieved an accuracy of 99.97 percent, which was slightly better than the performance of Simulation 2 because of the contribution from the semantic pathway, which allowed the network to distinguish the homographs. For nonword reading, the performance was 93.72% correct. On the stimuli from Weekes (1997) the network could correctly read all high-frequency words and low-frequency words. The performance on nonword reading was 91.6% correct. A 3 × 4 within-subjects ANOVA analysis was performed to analyse the error scores produced by the network. There were significant main effects for both word type, *F*(1.03, 9.23) = 679.63, *p* < .001 (with a Greenhouse–Geisser adjustment) and word length, *F*(3, 27) = 28.55, *p* < .001. There was also a significant word length by word type interaction, *F*(1.98, 17.84) = 18.73, *p* < .001 (with a Greenhouse–Geisser adjustment). Fig. 10 shows the mean error score at the phonological level as a function of word length and word type. The interaction pattern of the word length and word type is similar to that seen in Simulation 2 (Fig. 7). However, in Simulation 3 the difference between words and nonwords has increased. There was still a small but reliable length effect for high-frequency words *F*(1.27, 11.46) = 27.65, *p* < .001 (with a Greenhouse–Geisser adjustment). A linear regression analysis was performed to compare the regression coefficients for the different word types, the coefficient for nonwords (slope = .091; CI: .010–.172) was much greater than that for both low-frequency words (slope = .007; CI: 003–.012) and high-frequency words (slope = .004; CI: .002–.006), consistent with the findings in Simulation 2 of the differential word-length effects on naming words and nonwords. In addition, the regression coefficient for high-frequency words was much smaller than that in Simulation 2 (slope = .013; CI: .007–.018), showing that the length effect for high-frequency words was compressed by the addition of semantics.

## 6 Simulation 4: Other visual representations and serial effects

The simulations so far have clearly demonstrated that this parallel model can account for the length by lexicality interaction that had previously been thought to require a serial processing mechanism. However, before it can be truly regarded as a viable model of word naming it is important to investigate two additional issues: (1) To what extent do the results reported so far depend on the vowel-centred visual representation scheme that we have adopted. (2) Can the model be extended to account for other “serial” effects in reading, in particular the “position of irregularity effect” (Rastle & Coltheart, 1999) and “whammy effects” (Rastle & Coltheart, 1998).

### 6.1 Other types of visual representations

The visual representations used so far adopt a vowel-centred coding scheme, which we have argued is a close approximation to the optimal viewing position (OVP; McConkie et al., 1989; Oregan et al., 1984). However, the exact alignment of the OVP for any particular word is not precisely defined and may well vary between individuals or even between fixations in the same individual (Rayner, 1998). Since it remains unclear exactly how words are optimally positioned, it is important to explore how changes in word alignment would affect our pattern of results. To investigate this we trained the network under several different word alignment policies covering a range of possible OVP positions and including one version with variable positioning. The different policies were as follows:

1. Vowel-centred with variability of one position left or right (VowelLR): this included the original vowel-centred set together with two additional sets shifting one position to the left or right of the original fixated column.
2. Optimal viewing position 1 (OVP1): the words were fixated one position left of the centre.
3. Optimal viewing position 2 (OVP2): the odd-length words were fixated at the centre and others were fixated at the one position left of the centre.
4. Optimal viewing position 3 (OVP3): each word with less than 5 letters was fixated one position left of the centre and longer words were fixated at the third letter.
5. Left aligned (LA): each word was aligned on its first letter.

Very long words, which could not be accommodated by using 12 slots when shifting the words to one position right of the original fixated column, were removed. For the VowelLR version, which included three times as many stimuli as the original, the number of units in the first two hidden layers was increased to 100 and 200 respectively. All other details were the same as Simulation 2. The networks’ performance on naming words and nonwords is summarised in Table 4. All of the networks performed similarly to Simulation 2 regardless of the exact positioning of the visual stimuli with accuracy on words varying between 97.13 and 99.63, and nonword accuracy varying between 93.25 and 97.21.

In addition to testing accuracy on naming words and nonwords, it is important to examine whether the networks can produce both the frequency by consistency and the word length by lexicality interactions. The results for these tests are summarised in Tables 5 and 6. Regardless of the alignment of visual representations all networks produced the expected interactions of frequency by consistency (all *p*s < .05) and lexicality by length (all *p*s < .05), except for the left aligned (LA) version where the lexicality by length interaction was only marginally significant (*p* = .071).

Overall, these results demonstrate that simulation of the length by lexicality effect is not critically dependent on the exact positioning of the visual stimuli.

### 6.2 Simulations of other serial effects

In addition to the length by lexicality interaction there are two other ”serial” effects, which are also often used as evidence against parallel models: the position of irregularity effect (Rastle & Coltheart, 1999) and the whammy effect (Rastle & Coltheart, 1998). Rastle and Coltheart (1999) found a position of irregularity effect in reading low frequency exception words. The regularity effect was stronger for first position irregular words (e.g., chef) than for second position irregular word (e.g., cooks), and it was stronger for second position irregular word than for third position irregular words (e.g., glow). This effect has been interpreted as evidence for serial processing, which appears to be inconsistent with any model that maps orthographic representations to phonological representations in parallel. However, Zorzi’s (2000) CDP model (a fully parallel model) was able to capture this effect and he further found the position of irregularity factor was actually confounded with grapheme–phoneme consistency. In addition, Kawamoto, Kello, Jones, and Bame (1998) have argued that this effect might be explained by the nature of articulatory processes. The cost of irregularity observed for first position irregular word is likely to result from initiating articulation. For words with late irregularities, this effect is not obvious (but see Rastle, Harrington, Coltheart, and Palethorpe (2000) for a co-articulatory account).

Regarding to the whammy effect, Rastle and Coltheart (1998) demonstrated that 5-letter nonwords with 3 graphemes produced longer latencies than 5-letter nonwords with 5 graphemes because of the complexity of multi-letter graphemes. According to the serial processing account, the spurious phoneme unit will activate and then affect the late activation of the correct phoneme. However, the whammy effect may also be explained by the parallel processing account simply due to the competition between phonemes. In fact, Rastle and Coltheart (1998) and Coltheart et al. (2001) have tested the PMSP96 model on the same nonword stimuli and it showed a marginally significant whammy effect (*p* = .058). It seems that both effects are still somewhat contentious. Nevertheless it remains the case that whatever the theoretical source of these effects they are empirically valid. Thus, it is important to investigate whether the model can simulate them.

#### 6.2.1 Position of irregularity effects

The model was tested on the word stimuli used by Rastle and Coltheart (1999). The list included 88 low-frequency irregular words, and 88 low-frequency regular words matched on number of letters, initial phoneme and frequency. For the current simulation, two irregular words were excluded because they were not in the model’s vocabulary. The model achieved 98.98% and 98.02% accuracy on naming the regular and irregular words respectively. Error items were discarded from subsequent analyses along with any responses outside three standard deviations from the mean error scores. After those items were removed from analysis, 85 matched pairs of items remained.

A repeated measures ANOVA was performed on the mean error scores, with regularity and position as within-subject factors. The results showed that both the main effect of position, *F*(2, 18) = 67.92, *p* < .001, and the main effect of regularity, *F*(1, 9) = 185.76, *p* < .001, were significant. There was an significant interaction between regularity and position, *F*(2, 18) = 8.25, *p* = .003, as shown in Fig. 11a. Bonferroni post hoc comparisons of regularity groups showed that the irregular words produced significantly higher error scores than did regular words, *p* < .05. Results of position conditions showed that all pairwise comparisons were significant, all *p*s < .05.

The main point of this effect is to show the cost of irregularity for low-frequency exception words would be modulated by the position of irregularity. Fig. 11b shows the *Z* transformation of the mean error scores of the network on the irregular words across positions. To compare with human data, the corresponding item latencies in the Rastle and Coltheart (1999) study were used. The *Z* score obtained from the human naming data were plotted in Fig. 11b as well. In addition, networks with different visual representations were tested on this effect and their *Z* scores of the irregular words were included in the same figure. All of the patterns produced by the networks were very similar to those seen in the human data.

#### 6.2.2 Whammy effects

The vowel-centred version of the model was tested on two nonword sets consisting of 3 and 5 grapheme words as used by Rastle and Coltheart (1998). Some of these nonword stimuli are unusual because a number of items have unique orthographic bodies (e.g., elst). These items would not be processed correctly because the model will not have had the opportunity to learn the correct pronunciations of similar words, and so will have no basis for generalisation. Accordingly, these items were removed along with their pairwise matches. That left 18 matched pairs of items. A one way repeated measure ANOVA was performed on the error scores from these items. The result showed a significant “whammy” effect, *F*(1, 9) = 6.42, *p* = .032, with the 3-grapheme items producing higher error scores (*M* = 0.344 SD = 0.073) than the 5-grapheme items (*M* = 0.236 SD = 0.084).

## 7 General discussion

This paper describes a parallel distributed processing model of single word reading that includes a visual processing component. Simulation 1 used this component to map onto an orthographic layer with predetermined representations. Its performance on naming words and nonwords was similar to that achieved by PMSP96 (Plaut et al., 1996). In Simulation 2, the same network was trained without predefined orthographic representations. Overall performance was similar to Simulation 1 except when testing for word-length effects. Simulation 2 could account for the differential word-length effects observed in normal human reading, while Simulation 1 did not show the linear effect of length seen in human data. These data provide compelling evidence that the existence of a length by lexicality interaction does not force the conclusion that there is a serial processing component to reading. Simulation 3 showed that the addition of a semantic contribution to phonology, acts to increase the interaction between lexicality and length. The length effect for real words was still not completely eliminated leaving a length effect for high-frequency words that was reliable but smaller than in Simulation 2, and much smaller than the length effect for nonwords (see Fig. 10). These results provide evidence of the existence of small length effects for reading high-frequency words, which appears to be somewhat inconsistent with Weekes’ data. However, it might be reasonable to assume that Weekes’s failure to find any length effect for high frequency words was in fact due to a lack of statistical power as there has been subsequent evidence indicating that there should be a small length effect even for high frequency words (Balota et al., 2004; New et al., 2006; Penolazzi et al., 2007). Perhaps the best interpretation of the available data is that there is an interaction between word lexicality and word length such that the word-length effects for nonwords are greater than low-frequency words (Richards & Heller, 1976; Weekes, 1997), and the length effects for low-frequency words are greater than high-frequency words (Balota et al., 2004; Penolazzi et al., 2007; Weekes, 1997). Our findings are support this interpretation. In addition, Simulation 4 demonstrated that these results are not dependent upon the exact choice of fixation position, and that they can be extended to account for other ”serial” effects such as position of irregularity and whammy effects.

The differences between Simulation 1 and Simulation 2 may be attributable to the way in which the dispersion problem is tackled by the two networks. Following Plaut et al. (1996) Simulation 1 adopted the same coding for both orthographic and phonological representations. This coding scheme completely removes any trace of dispersion between different letter positions in each individual segment of the word (onset, vowel and coda). However, this scheme is clearly not completely realistic from a biological point of view as orthographic representations have to be developed as part of the process of learning to read and unlike phonological representations are not predefined by earlier language learning. To address this problem Simulation 2 was allowed to develop its own orthographic representations. It is important to emphasise that Simulation 2 did have a stage for orthographic processing but orthography is implemented in the form of an intermediate layer of hidden units mediating visual information and generating inputs for the later stage of phonological processing. This led to an emergent length effect which was greater for nonwords than for words. Nonword reading accuracy was also closer to human performance for Simulation 2 indicating that the use of structured phonological representations on their own are sufficient to guide the development of a set of orthographic representations that alleviate the dispersion problem. From this it seems clear that the emergent lexicality by length interaction stems from the pressure to learn the mappings between visual representations, which are strongly influenced by letter position and central phonological representations that are independent of length, but constrained by phonotactic considerations. If this is true then we might expect that Simulation 1 would also show a length by lexicality interaction, but here it would be hidden in the mappings between the visual and orthographic layers. Interestingly, the error patterns of the orthographic output in Simulation 1 do in fact show a lexicality by length interaction similar to that produced by Simulation 2 (see Fig. 4b). Of course the orthographic coding that emerges in Simulation 2 may not be as perfect as the one used in Simulation 1 and PMSP96 in terms of overcoming the dispersion problem; however, it does seem more likely to resemble to the scheme adopted in human reading system because the performance of Simulation 2 on nonword reading is closer to that of human subjects; and importantly Simulation 2 can replicate the differential word-length effects observed in human subjects.

If our model is correct and length effects are an emergent property of visual processing then there should be convergent evidence from imaging studies that show length effects and a length by lexicality interaction in visual processing areas. Two recent studies provide just such data: An MEG study conducted by Wydell et al. (2003) found early length effects for both words and nonwords in the occipital cortex. They further found a length by lexicality interaction of the cortical activations in the posterior left superior temporal cortex. A more recent fMRI study by Schurz et al. (2010) also showed a substantial length effect on activation for both words and nonwords in the left occipital cortex and identified the VWFA as exhibiting an interaction of length by lexicality. These findings suggest that visual processing is involved in the emergence of length effects in normal reading. These data are therefore entirely consistent with our hypothesis. In our model, the visual layer could be considered as a visual processing component corresponding to primary visual cortex with the orthographic layer mapping onto the VWFA.

The key result of our study is that an interaction between length and lexicality can arise from a single mechanism in a parallel model. Previously Weekes (1997) argued that his data implied that there must be a serial component to reading. This was on the basis that the length effect for nonword reading reflects the sequential operation of a non-lexical reading mechanism. This interpretation supports models of reading that assume a serial reading mechanism for nonwords such as the DRC or CDP+ models. Note:although the CDP+ model is a parallel model it has a specific serial component for parsing graphemes, which generates the interaction between length and lexicality. Perry et al. (2007) tested a completely parallel CDP+ model (i.e., without a serial graphemic parsing process) on Weekes’ data and the result showed no trace of a length effect. The current simulation results clearly demonstrate that a fully parallel model with a visual processing stage can produce a significant interaction between word length and word lexicality.

Both the position of irregularity effect and the whammy effect have been taken, along with the length effect, as evidence of serial processing, and against parallel models (Coltheart et al., 2001; Perry et al., 2007; Rastle et al., 2009). However, there are convincing alternative accounts for both of these effects. The position of irregularity effect is complicated by other factors such as grapheme to phonology consistency (Zorzi, 2000) and frequency (Perry et al., 2007). Thus, one may not be compelled to interpret it as a serial effect. In fact both the parallel CDP model and the present parallel model can simulate this effect, which strongly suggests that the alternative account may be correct. With regard to the whammy effect, the present parallel model simulates the effect, clearly demonstrating that it cannot be used to arbitrate between serial and parallel models. This suggests the competition between multiple graphemes is sufficient as an alternative explanation for the origin of this effect in humans. It is interesting to note that although these effects are often taken together as evidence of serial processing, our modelling suggests a dichotomy in that the whammy and serial position effects arise from consistency and frequency whereas the length by lexicality effect arises from the nature of the mapping between visual and phonological representations and requires a model with a visual processing system to fully capture it.

The role of visual processing in accounting for the length effect is particularly interesting in view of the neuropsychological evidence from pure alexia patients. The hallmark of pure alexia is an abnormally strong length effect which is thought to result from a visual deficit. The current reading model would serve as a good opportunity to investigate the possibility of modelling length effects in PA patients.

In summary, these simulation results suggest that the key to understanding the emergence of length effects in parallel models is the transformation between visual representations, which are strongly influenced by letter position and word length, and central phonological representations that are independent of length, but constrained by phonotactic considerations. These results provide new evidence to challenge the assumption that a serial mechanism is required to account for length effects in word and nonword reading.

## Figures and Tables

**Fig. 4a:**
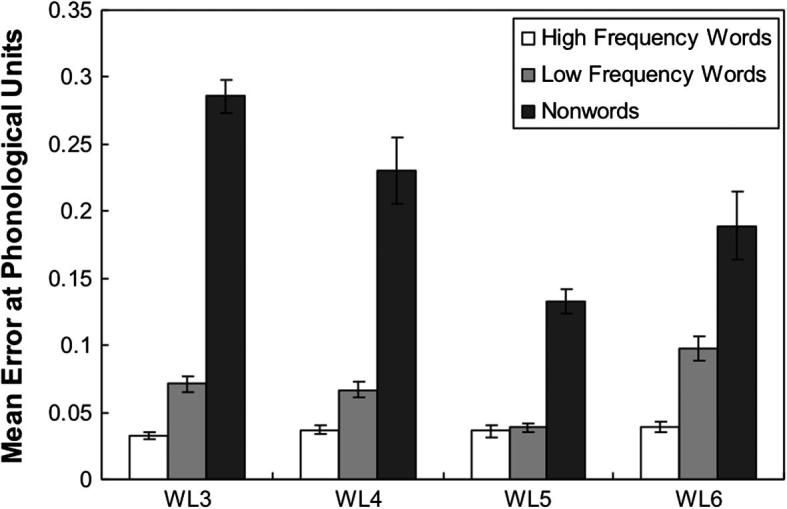
Mean error score computed at the phonological level for Simulation 1 as a function of word length and word type. Error bars represent standard errors.

**Fig. 4b:**
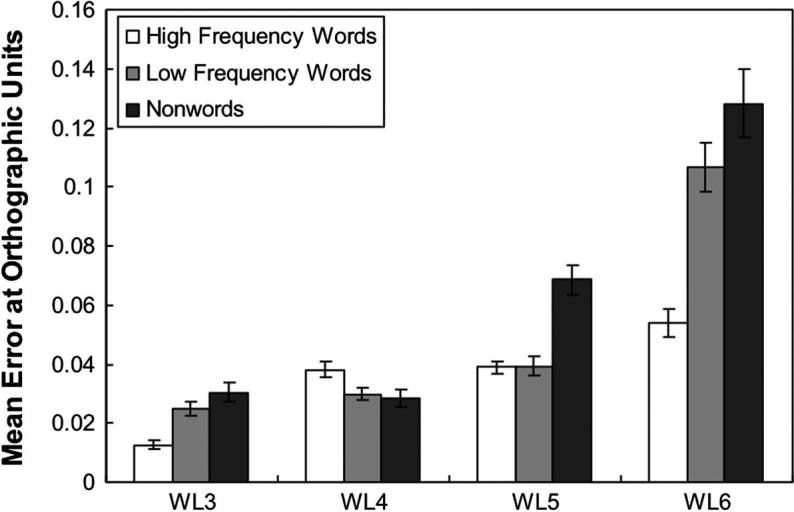
Mean error score computed at the orthographic level for Simulation 1 as a function of word length and word type. Error bars represent standard errors.

**Fig. 9a:**
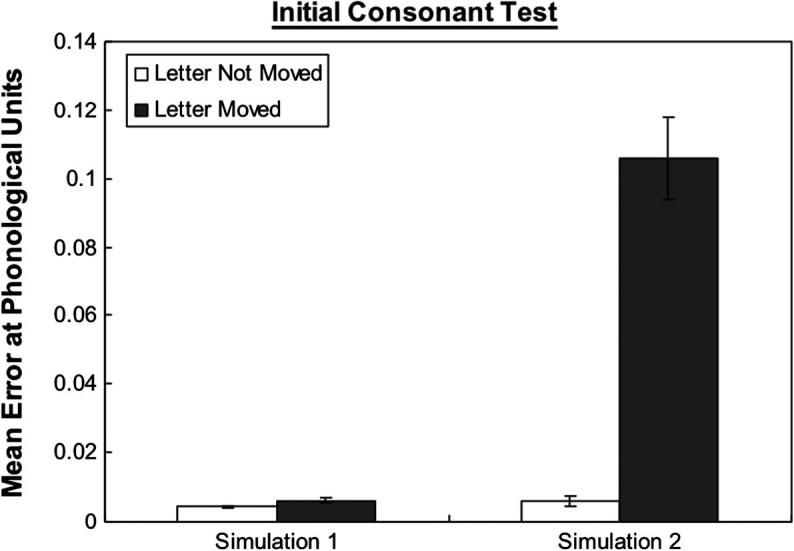
Mean error score of the networks on 3-letters words with the initial consonant in moved or not moved condition. The error score was for the individual letter rather than for whole word. Error bars represent standard errors.

**Fig. 9b:**
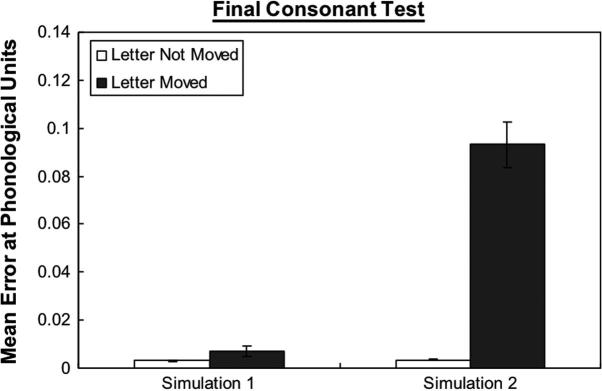
Mean error score of the networks on 3-letters words with the final consonant in moved or not moved condition. The error score was for the individual letter rather than for whole word. Error bars represent standard errors.

**Fig. 11a:**
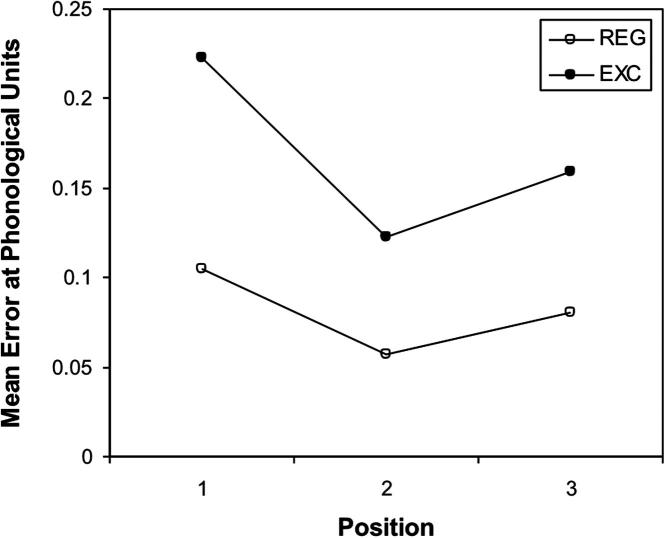
Mean error score computed at the phonological level for Simulation 2 on the regular and irregular words as a function of irregularity position.

**Fig. 11b:**
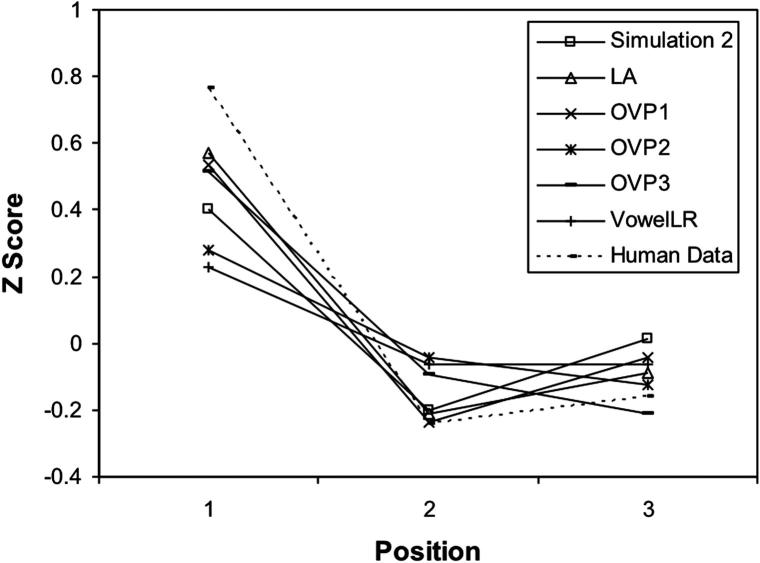
Z transformation of mean error scores from the networks with different visual representations plotted against human data for reading irregular words. Human Data is taken from Appendix D in the Rastle and Coltheart (1999) study.

**Table 1:** Orthographic and phonological representations.

*Orthography*	
Onset	Y S P T K Q C B D G F V J Z L M N R W H CH GH GN PH PS RH SH TH TS WH
Vowel	E I O U A Y AI AU AW AY EA EE EI EU EW EY IE OA OE OI OO OU OW OY UE UI UY
Coda	H R L M N B D G C X F V J S Z P T K Q BB CH CK DD DG FF GG GH GN KS LL NG NN PH PP PS RR SH SL SS TCH TH TS TT ZZ U E ES ED

*Phonology*	
Onset	sS C z Z j f v T D p b t d k g m n h l r w y
Vowel	a e i o u @ ^ A E I O U W Y
Coda	r l m n N b g d psksts s z f v p k t S Z T D C j

**Table 2:** Performance of the network on words and nonwords (difference from human subjects in brackets).

	Current Training Corpus	Plaut et al. (1996)	Glushko (1979)
	Words	Words	Consistent Nonwords
Human subjects	99.84^a^	99.56^a^	93.80
PMSP96 model	–	99.13 (−0.43)	97.70 (3.90)
Simulation 1 (orthography)	99.26	99.57	99.77
Simulation 1 (phonology)	99.06 (−0.78)	99.07 (−0.49)	98.14 (4.34)
Simulation 2 (phonology)	99.19 (−0.65)	99.13 (−0.43)	93.25 (−0.55)
Simulation 3 (phonology)	99.97 (0.13)	99.96 (0.40)	93.72 (−0.08)

a This is estimated upper bound based on the number of homographs in the corpus. It is assumed that all words would be pronounced correctly and that homographs would be correct at a certain percentage of the time based on chance level performance.

**Table 3:** The probability of location of individual letter within a word.

	L-Pos5	L-Pos4	L-Pos3	L-Pos2	L-Pos1	V-Pos	R-Pos1	R-Pos2	R-Pos3	R-Pos4	R-Pos5	R-Pos6
A	0	0	0	0	0	0.7966	0.1995	0.0039	0	0	0	0
B	0	0	0	0.2347	0.5472	0	0.1418	0.0762	0	0	0	0
C	0	0	0.0044	0.2786	0.2676	0	0.2515	0.1799	0.0180	0	0	0
D	0	0	0	0.0443	0.1219	0	0.1095	0.2123	0.1593	0.3076	0.0432	0.0020
E	0	0	0	0	0	0.3633	0.1011	0.3006	0.2049	0.0291	0.0010	0
F	0	0	0	0.2138	0.4324	0	0.2209	0.1329	0	0	0	0
G	0	0	0	0.1913	0.2086	0	0.2407	0.3454	0.0140	0	0	0
H	0	0	0	0.0398	0.6196	0	0.0052	0.1368	0.1788	0.0189	0.0009	0
I	0	0	0	0	0	0.8109	0.1891	0	0	0	0	0
J	0	0	0	0	0.9960	0	0.0040	0	0	0	0	0
K	0	0	0	0.0600	0.1135	0	0.1460	0.6759	0.0047	0	0	0
L	0	0	0	0	0.4402	0	0.2777	0.2712	0.0105	0.0004	0	0
M	0	0	0	0	0.4259	0	0.3749	0.1952	0.0041	0	0	0
N	0	0	0	0	0.1649	0	0.5744	0.2468	0.0139	0	0	0
O	0	0	0	0	0	0.8941	0.1034	0.0025	0	0	0	0
P	0	0	0.0030	0.1904	0.3535	0	0.2380	0.2148	0.0003	0	0	0
Q	0	0	0	0.5659	0.2908	0	0.0666	0.0766	0	0	0	0
R	0	0	0	0.0030	0.4963	0	0.3435	0.1305	0.0248	0.0019	0	0
S	0	0.0005	0.0444	0.1991	0.0990	0	0.1488	0.1685	0.2881	0.0464	0.0050	0.0001
T	0	0	0.0188	0.1981	0.2089	0	0.2103	0.2615	0.0833	0.0185	0.0006	0
U	0	0	0	0	0.0396	0.6213	0.3132	0.0142	0.0116	0	0	0
V	0	0	0	0	0.2624	0	0.5026	0.2350	0	0	0	0
W	0	0	0	0.1619	0.4687	0	0.3594	0.0101	0	0	0	0
X	0	0	0	0	0	0	0.8983	0.0778	0.0239	0	0	0
Y	0	0	0	0	0.2583	0.2626	0.4791	0	0	0	0	0
Z	0	0	0	0	0.2063	0	0.4095	0.3198	0.0645	0	0	0

*Note*: V-Pos: the position of the first vowel of each word; L-Pos*K*: *K* position(s) left of V-Pos; R-Pos*K*: *K* position(s) right of V-Pos.

**Table 4:** Performance with different types of visual representations.

Network type	Training corpus	Glushko (1979) Consistent Nonwords
Simulation 2	99.19	93.25
VowelLR	97.13	94.81
LA	99.30	93.72
OVP1	99.45	94.19
OVP2	98.73	93.95
OVP3	99.63	97.21

*Note*: VowelLR: vowel centred with the variability of one position left or right; LA: left aligned; OVP1: optimal viewing position 1; OVP2: optimal viewing position 2; OVP3: optimal viewing position 3.

**Table 5:** ANOVA results of the networks with different types of visual representations on the frequency and consistency effect.

Network Type	Main effect				Interaction	
	Frequency		Consistency		Frequency × consistency	
	*F*(1, 92)	*P*	*F*(1, 92)	*P*	*F*(1, 92)	*P*
VowelLR	41.57	<.001	100.95	<.001	28.73	<.001
LA	31.28	<.001	50.84	<.001	12.18	=.001
OVP1	29.46	<.001	43.80	<.001	18.52	<.001
OVP2	50.51	<.001	80.71	<.001	31.97	<.001
OVP3	42.19	<.001	65.72	<.001	26.92	<.001

*Note*: VowelLR: vowel centred with the variability of one position left or right; LA: left aligned; OVP1: optimal viewing position 1; OVP2: optimal viewing position 2; OVP3: optimal viewing position 3.

**Table 6:** ANOVA results of the networks with different types of visual representations on the word length and lexicality effect.

Network type	Main effect				Interaction	
	Length		Lexicality		Length × lexicality	
	*F*(3, 27)	*P*	*F*(2, 18)	*P*	*F*(6, 54)	*P*
VowelLR	23.19^a^	<.001	573.50	<.001	6.04	<.001
LA	14.57	<.001	191.72^a^	<.001	2.74^a^	=.071
OVP1	11.96	<.001	173.28^a^	<.001	4.94^a^	=.014
OVP2	19.12	<.001	175.91^a^	<.001	4.95^a^	=.012
OVP3	7.18	=.001	179.75^a^	<.001	5.01^a^	=.017

*Note*: VowelLR: vowel centred with the variability of one position left or right; LA: left aligned; OVP1: optimal viewing position 1; OVP2: optimal viewing position 2; OVP3: optimal viewing position 3.

a The value was with a Greenhouse–Geisser adjustment to compensate for the violation of sphericity assumption.
